# Axolotls retain fertility throughout lifespan

**DOI:** 10.1186/s12915-026-02545-3

**Published:** 2026-02-14

**Authors:** Yuliia Haluza, Beate Gruhl, Anja Wagner, Thomas Kurth, Elly M. Tanaka, Maximina H. Yun

**Affiliations:** 1 Center for Regenerative Therapies Dresden (CRTD), Dresden University of Technology, Dresden, Germany; 2 Core Facility Electron Microscopy and Histology Facility, Technology Platform, Center for Molecular and Cellular Bioengineering (CMCB), Dresden University of Technology, Dresden, Germany; 3 Institute of Molecular Biotechnology of the Austrian Academy of Sciences (IMBA), Vienna BioCenter (VBC), Vienna, Austria; 4 Chinese Institutes for Medical Research (CIMR), Beijing, China; 5 Physics of Life (PoL), Cluster of Excellence, Dresden University of Technology, Dresden, Germany

**Keywords:** Reproductive ageing, Salamander, Ovary, Regeneration, Atresia

## Abstract

**Background:**

Salamanders such as axolotls exhibit exceptional regenerative abilities and longevity. While many ectothermic species reproduce into old age, axolotls have been proposed to experience post-maturation fertility decline.

**Results:**

We hereby present a large-scale assessment of axolotl reproductive potential across lifespan based on over 15 years of mating records from captive breeding. We show that axolotl egg number, egg quality, and mating success rates peak after sexual maturation and gradually decline up to 4 years of age, with rates stabilising after the early-life maturation period. We also report that axolotls preserve early-stage oocytes until advanced age and describe the progression of follicular atresia in salamanders.

**Conclusions:**

By breeding older individuals, we show that axolotls retain functional fertility until ages within their average lifespan, exhibiting limited reproductive senescence. This study offers insights of relevance to developmental and ageing studies and provides a comparative model for understanding how long-lived vertebrates maintain reproductive capacity and support longer survival through time.

**Supplementary Information:**

The online version contains supplementary material available at 10.1186/s12915-026-02545-3.

## Background

Ageing leads to organismal changes characterised by molecular and cellular hallmarks that both reflect and promote this process [1]. These changes manifest as increased mortality and reduced fertility, key aspects of physiological ageing, seen throughout the animal kingdom [2–4]. In mammals, a gradual reproductive decline is characterised by reduced gamete quality and number, endocrine dysregulation, and deteriorative changes in reproductive organs [5]. Despite rare incidences of reproductive senescence in fish [6, 7], amphibians (reviewed in [8]), and reptiles [9], reproductive output in ectothermic vertebrates often does not decline with age [10–12].

Despite its potential to offer insights into the preservation of fertility and longevity, salamander reproductive ageing remains largely unexplored. Salamanders such as axolotls (*Ambystoma mexicanum*) are widely known for their remarkable regenerative capabilities. They are able to regenerate a number of injured body parts including limbs, spinal cord, lungs, jaws, and sections of heart and brain without any resulting functional impairment [13, 14]. Furthermore, axolotls also demonstrate an exceptional resistance to age-related diseases, including cancer, and show few physiological signs of decline even at an advanced age [15, 16].

Notably, their regenerative potential extends to ovary regeneration. Even in adulthood, axolotls retain a reserve of oogonial stem cells that, similar to spermatogonial stem cells, remain mitotically active and continuously contribute to oogenesis [17]. This sustained capacity for germ cell renewal highlights their potential for prolonged reproductive activity. Following egg fertilisation, axolotl embryogenesis lasts approximately 2 weeks. After hatching, larvae continue to develop and rapidly grow, with individuals reaching sexual maturity at around 9–12 months old [18]. Axolotls regularly lay hundreds of eggs per clutch [19] and are reported to produce eggs for years [17]. However, it has been proposed that axolotls exhibit age-related decline in fertility [18]. Among these are anecdotal observations suggesting fertility declines after the early years for both males and females yet more pronounced in the latter [18]. Such declines have not been observed for other salamander species [16, 18].

Studying reproductive ageing in amphibians is further challenged by the complexity of achieving successful breeding in captivity [20, 21]. The existence of long-term axolotl colonies has overcome this constraint, enabling the keeping of mating records over several years and thus the analysis of reproductive potential across ageing under controlled conditions.

Here, we provide an in-depth analysis of mating and reproductive success in axolotls and quality assessment of produced eggs, highlighting overall high fertilisation rate in animals below 4 years of age. We find that axolotls are able to mate repeatedly and independently of seasonality without further stimulation. Further, we uncover sex-related differences, with egg quality declining with female age and mating success with male age. We show that the oocyte pool is preserved over time, albeit with a mild decrease after the first 2 years of life. The initial decline in egg number and quality and increased rate of unsuccessful matings gradually plateaus as individuals approach 4 years of age. Finally, by breeding older individuals, we demonstrate that axolotls retain fertility up to ages within their average lifespan.

## Results

### Axolotl display a 49% mating success rate in captive breeding conditions

To characterise the efficiency of axolotl matings, we analysed over 15 years of reproductive records from an axolotl colony first established at the Max Planck Institute of Molecular Cell Biology and Genetics and later operated at the CRTD (Center for Regenerative Therapies Dresden) (records corresponding to the Tanaka lab—until 2017—and to the Yun lab—from 2017 onwards; ‘ Methods’ [22]). These records included information on the male and female used for each mating, the date of mating, and the dates of visible spermatophore and egg deposition. Matings were classified as successful if both events occurred. In unsuccessful matings, neither spermatophores nor eggs were laid.

**Fig. 1:**
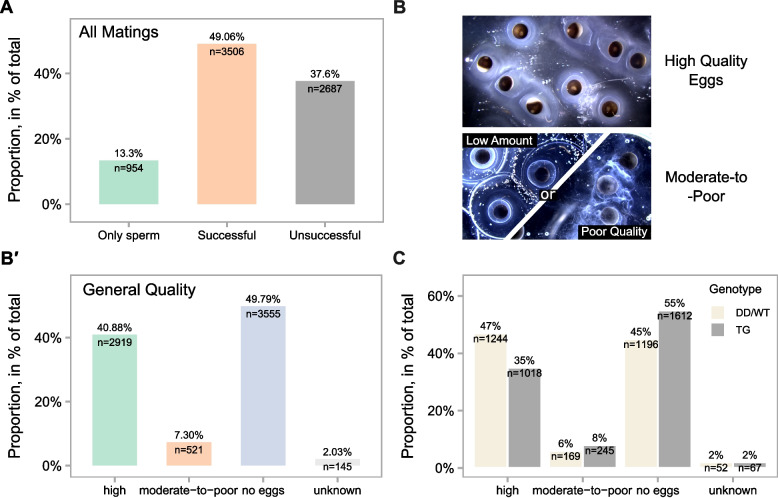
Axolotls exhibit a 49% mating success rate in captive breeding.** A** Axolotl colony mating statistics, shown as percentage (*y*-axis). **B** Visualisation of eggs by quality categories. Low amount denotes morphologically normal eggs with a clear jelly layer but in low numbers (< 150 eggs per mating). Poor quality denotes morphologically abnormal eggs with reduced developmental potential and a loose jelly. **B′** Variation in egg quality (*x*-axis) across matings, shown as percentage (*y*-axis). **C** Egg quality (*x*-axis) differences between wild-type axolotls and transgenic lines. Percentages (*y*-axis) show proportions within respective genotype groups. *n* in **A**, **B′**, and **C** denotes number of matings

Remarkably, in captive breeding conditions, axolotls exhibited a 49% reproductive success rate (Fig. 1A). In 13% of cases, only spermatophores were deposited, indicating that courtship interactions are not always effective, and females may not be receptive to that male. Such behavioural aspects are crucial in understanding the effects of reproductive ageing. Additionally, we observed that c. 37% of matings were unsuccessful, with neither spermatophores nor eggs observed (Fig. 1A). Seasonal analysis showed consistent success rates throughout the year, with minimal variation (≤ 3%) between quarters (Additional file 1: Fig. S1A).

When a mating is successful, axolotls regularly lay hundreds of jelly-coated eggs. Yet, not all eggs are fertilised or develop successfully (Additional file 1: Fig. S1B, 1–2), suggesting variation in reproductive outcomes. These outcomes are closely linked to the mating process, which begins with the male performing a courtship dance and depositing spermatophores (Additional file 1: Fig. S1B, 3) that the female may pick up for internal fertilisation. Successful mating events may result in eggs of variable quality, based on viability, morphology, and/or jelly layer characteristics (Fig. 1B). For our analysis, we defined ‘high-quality eggs’, distinguished by a high total egg count, a high fertilisation success, and the production of viable embryos. The remaining ones were classified as ‘moderate-to-poor quality eggs’, characterised by fewer than 50% being viable and fertilised, a loose jelly coat, and a low total egg count (Fig. 1B, detailed description in ‘ Methods’). This assessment results from long-term experience and is based on subjective observations. Notably, matings resulting in high-quality eggs accounted for almost 41% (Fig. 1B**′**). In contrast, 50% of pairings resulted in no eggs laid, and around 7% in moderate-to-poor quality eggs (2% unknown). These observations are consistent with previously published records from the Ambystoma Genetic Stock Center. In particular, breeding 13 axolotl pairs resulted in an approximate 60% mating success rate [23].

It is worth noting that wild-type (WT) or white (DD) strain axolotls show slightly higher reproductive quality compared to transgenic lines (Fig. 1C, excluding knockout lines), with 47% of high-quality eggs observed in WT/DD animals compared to 35% in transgenic axolotls. Moreover, transgenic animals also exhibited a higher rate of no-egg events.

Collectively, these findings suggest that unlike breeding events in anurans, many of which exhibit a high failure rate to produce eggs under standard conditions [20, 21], matings in axolotls (WT/DD) in captive breeding are both efficient and reliable.

### Subtle declines in egg output with time reflect a marginally stronger male influence

**Fig. 2:**
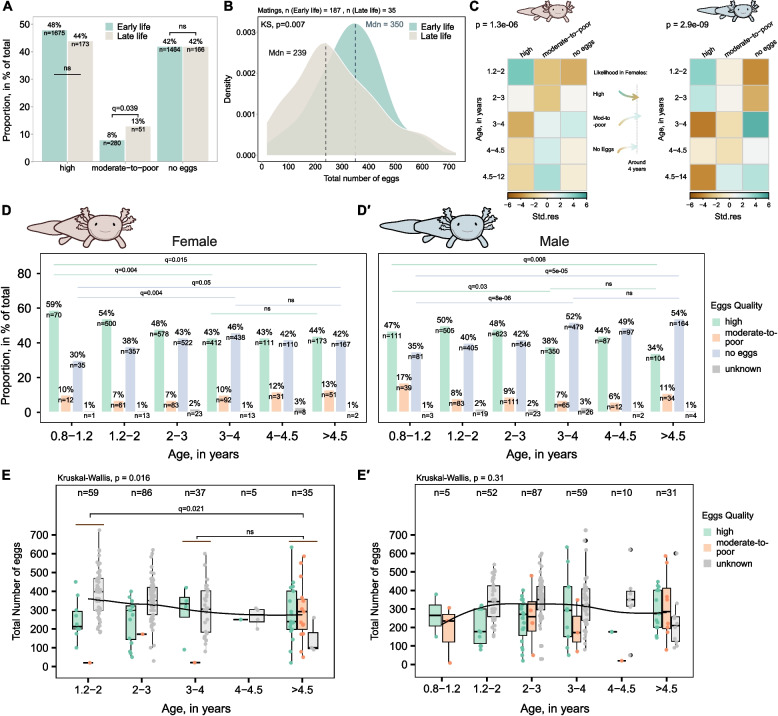
Egg quality and number subtly decline during early life.** A** Variation in egg quality (*x*-axis) by female age groups: early (≤ 4.5 years old) and late (> 4.5 years old) life. Unknown values are omitted. Percentages (*y*-axis) show proportions within respective age groups. **B** Distribution of egg number by female age group: early (≤ 4.5 years old) and late (> 4.5 years old) life. Curves show smoothed estimates of how the egg counts are distributed. Heights reflect relative frequency. Dashed line indicates the median egg number per age group. The difference between distributions was evaluated by Kolmogorov–Smirnov (KS) test, and *p*-value is indicated. Colours in **A** and **B** denote the age group. **C** Chi-squared test for categorical — egg quality versus age factor — observations in females (left, *χ*^2^ = 42.18, *p* = 1.3e-06) and males (right, *χ*^2^ = 55.91, *p* = 2.9e-09), highlighting the significant relationship between egg quality and age. Standardised residuals from the chi-squared test are reported, indicating the strength of deviation from expected egg quality for each age group. Positive values (in turquoise) represent higher-than-expected events, and negative values (in brown) represent lower-than-expected events. **D**, **E′** Variation in egg quality and number by female (**D** and **E**) and male (**D′** and **E′**) age groups. *y*-axis in **D** and **D′** denotes proportions within respective age groups (*x*-axis). *y*-axis in **E** and **E′** denotes egg numbers within respective age groups (*x*-axis). Colours in **D** and **E′** denote the egg quality category. Horizontal line in **E** and **E′** shows the trend in median egg number across age. Statistical significance of quality proportion differences between age groups in **A** and **D** and **D′** was evaluated by proportion test with Bonferroni correction. Statistical significance of median egg number difference among age groups in **E** and **E′** was evaluated by Kruskal–Wallis test and pairwise Dunn’s tests with Bonferroni correction, and *p*-values and *q*-values are indicated. *n* in **A**, **B**, **D**, and **D′** denotes number of matings while in **E** and **E′** the total number per age group

Our mating records allowed us to assess age-related trends in axolotl reproduction. We included both WT/DD and transgenic animals (excluding knockout lines) since in axolotl colonies matings with transgenics often outnumber wild-type strains (Additional file 1: Fig. S1C). The egg quality did not differ significantly between axolotls younger than 4.5 years (hereafter ‘early-life’ period, previously introduced in [24]) and animals older than 4.5 years (the ‘late-life’ period) (Fig. 2A). Late-life females showed a slight decrease in laying high-quality eggs, just a 4% difference compared to early-life females, and a 5% significantly higher rate of moderate-to-poor quality eggs (Fig. 2A). Matings yielding no eggs did not differ between both groups. However, it is important to note that mating statistics for younger animals include more entries compared to older females. This discrepancy may be explained by the fact that older animals started to be kept after 2017, and their matings—being rarer—were not as well-recorded. Further, we observed significant differences in total egg count distributions between late- and early-life axolotls (*p* = 0.007, Fig. 2B). In particular, the median number of eggs per mating decreased from 350 in early-life females to 239 in late-life females. In comparison, a previous study reported that young axolotls produced an average of around 250 eggs [23]. Building on this observation, we further investigated the role of age in shaping axolotl reproductive outcome. We selected smaller age groups with close to 1-year difference, except for the 4–4.5-year-old group (near the previously presumed age of fertility loss) and above 4.5-year-old group (due to limited sample size), and we also stratified the data by sex. We initially excluded 0.8–1.2 years old group (sexually maturing animals) from the analysis, as early matings may produce poorer quality eggs due to non-age-related factors. Applying a chi-squared test to determine the relationship between egg quality and age (Fig. 2C), we noted a significant association in both females (*χ*^2^ = 42.18, *p* = 1.3e-06) and males (*χ*^2^ = 55.91, *p* = 2.9e-09). High-quality eggs become less likely when either female or male axolotls older than 3 years are involved, with male age exerting a stronger effect (note the shift towards negative standardised residuals after 3 years, Fig. 2C). However, the likelihood of high-quality-egg matings stabilises around this age. In contrast, moderate-to-poor quality eggs are expected to be more frequently observed if females above 3 years old are mated, with a weaker male influence. The strongest association occurs in no-egg matings, with significantly higher frequencies observed in males over 3 years (Fig. 2C).

These observations were consistent with the exact proportions of egg quality across narrow age groups (Fig. 2D, D**′**). Following sexual maturation, females exhibit a gradual decline in the production of high-quality eggs, which stabilises around 3–4 years of age (Fig. 2D). Even at later life stages, approximately 44% of eggs remain high quality, indicating a sustained reproductive capacity. A similar dynamic is observed in males, where the percentage of high-quality eggs resulting from their matings decreases progressively, reaching a minimum of 33% with males over 4.5 years (Fig. 2D**′**). Evidently, the incidence of no-egg events increases with female age, plateauing around 3–4 years (Fig. 2D). On the contrary, the proportion of no-egg events in males continues to rise beyond 4.5 years of age (Fig. 2D**′**). Lastly, the proportion of moderate-to-poor-quality eggs also exhibits an age-related increase in females, a trend that is somewhat more pronounced than that observed in males.

Egg number varied mildly across age groups (Fig. 2E, E**′**), with a subtle decline in females and an apparent stabilisation around 3–4 years of age (Fig. 2E). Similarly, no strong effect of male age on egg number was detected (Fig. 2E**′**). This indicates that under the given captive breeding conditions, egg production is largely maintained with age. However, a reduction in egg quality emerges beyond 2 years of age alongside an increased likelihood of no-egg matings.

### Oocyte quality is maintained through lifespan despite a mild decline in the ovarian reserve until 3 years of age

**Fig. 3:**
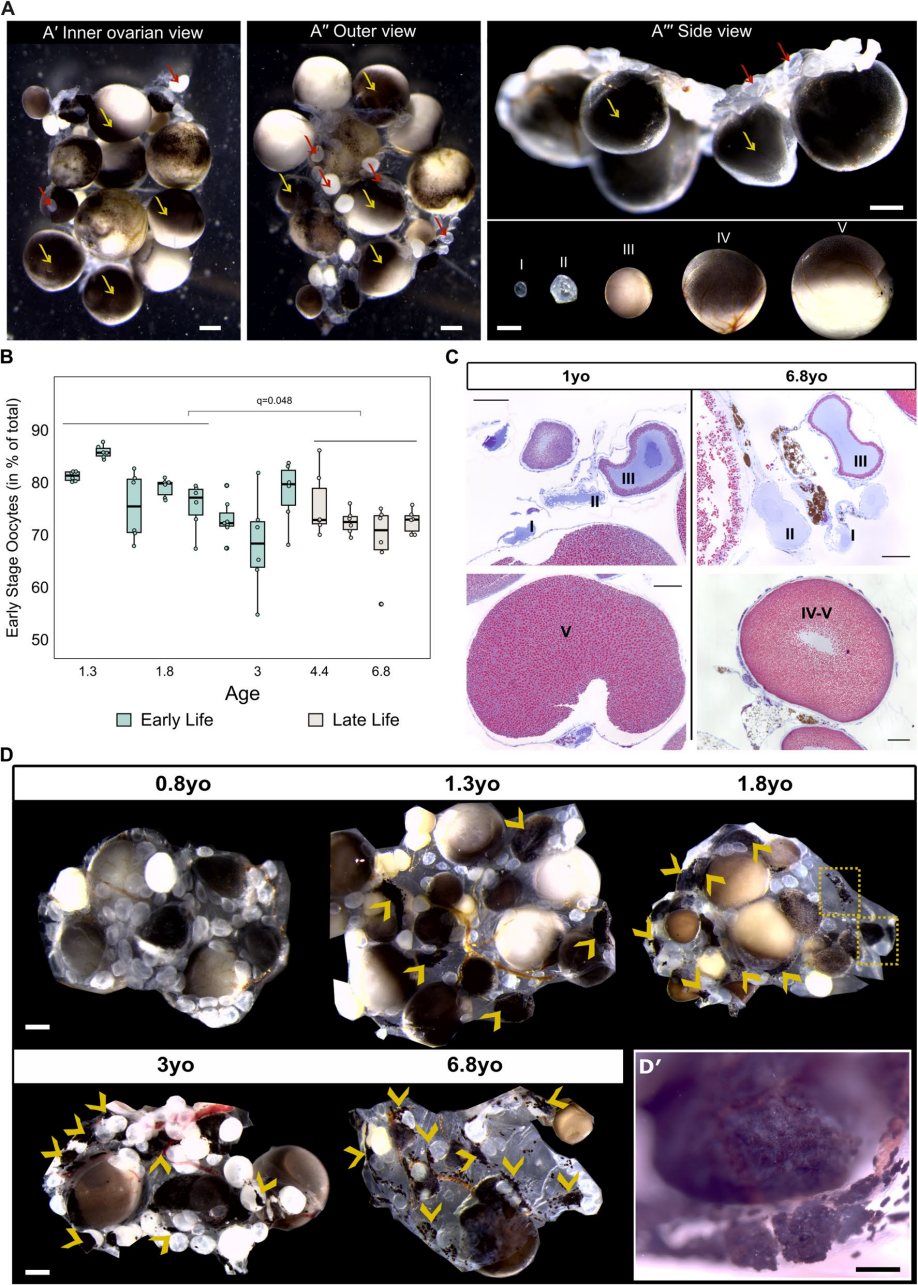
Mild decline in ovarian reserve in axolotls during the first 3 years of life while preserving function.** A** Representation of axolotl ovary. **A′** Inner ovarian view. **A″** Outer edge view. **A′′′** Above, side view; below, follicles containing oocytes at different maturation stages. Arrows: Red indicates early stage (I–III) and yellow — late stage (IV–VI). Note: Early-stage follicles (late II–III) 2–3 appear brighter under the microscope; it may result from increased light refraction and scattering due to yolk accumulation during vitellogenesis. Scale bar: 500 μm. **B** Early-stage follicles quantification across age (classified based on oocyte stages). Each box corresponds to one animal. Six samples were analysed per animal (*n* = 6). Statistical significance between early and late-life groups was evaluated by Wilcoxon test; *q*-value is indicated. Box plots centre line reports median. **C** Mallory trichrome-stained sections of 1- and 6.8-year-old ovarian tissue. Oocyte stages are denoted using Roman numerals. Scale bar: 200 μm. **D** Representative images of ovarian tissue from axolotls of varying ages. The age is indicated above the corresponding microscopy image. Scale bar: 500 μm. Arrows and dashed segments highlight atretic follicles. **D′** Close-up image of advanced stage atretic follicle. Scale bar: 200 μm

To investigate whether the decline in egg number observed in late-life axolotls stems from a depleted oocyte reserve, we next assessed the ovarian pool and examined age-related changes in the ovarian morphology (Fig. 3). As described previously, oocytes in axolotl span from stage 0 (oogonia to diplotene) to stage VI (mature oocytes) [17], in which maturing oocytes accumulate yolk platelets and pigments and grow extensively (Fig. 3A, A**′′′**). For the overview, we employed a whole-mount approach to examine the proportion of early-stage oocytes versus late-stage in sexually mature animals and up to late-life period (Additional file 1: Fig. S2, Fig. 3B). While what we observe microscopically are follicles, the classification is based on the developmental stage of the enclosed oocyte. For practical purposes and to facilitate interpretation, we considered oocytes stages I–III as early stage and those at stages IV–VI as late stage. Due to their small size and indistinct morphology, stage 0 oocytes (primary oogonia to postmitotic oocytes) could not be reliably identified in whole-mount preparations and were therefore excluded from the analysis. We observed that early-stage oocytes make up a large proportion of the ovarian pool, though their proportion declines significantly in late-life axolotls. Despite that, even in individuals of nearly 7 years of age, over 70% of oocytes were found to be at early stage (Fig. 3B), with the decline stabilising around 3–4 years of age. The ability to preserve early-stage oocytes even in older animals is presumably linked to their ovarian regeneration ability [17].

Histological analysis did not reveal any major alterations in oocyte morphology or the structure of the follicular envelope. Oocytes of all maturation stages were present (Fig. 3C). Cells with morphology consistent with germ cells were observed (histological assessment, Additional file 1: Fig. S3A [17]), though confirmation by immunostaining is further required. Age-related changes were characterised by the occasional presence of dark pigmented structures (Fig. 3C) and connective tissue expansion with possible collagen deposition (Additional file 1: Fig. S3B, B′), which stained intensely blue with Mallory’s trichrome. Changes were also evident in whole-mount ovary samples, where we observed a gradual accumulation of irregularly shaped, pigment-laden structures (Fig. 3D, D**′**). While these features were already detectable in young sexually mature animals (1.3 years old), they became more noticeable with advancing age.

**Fig. 4:**
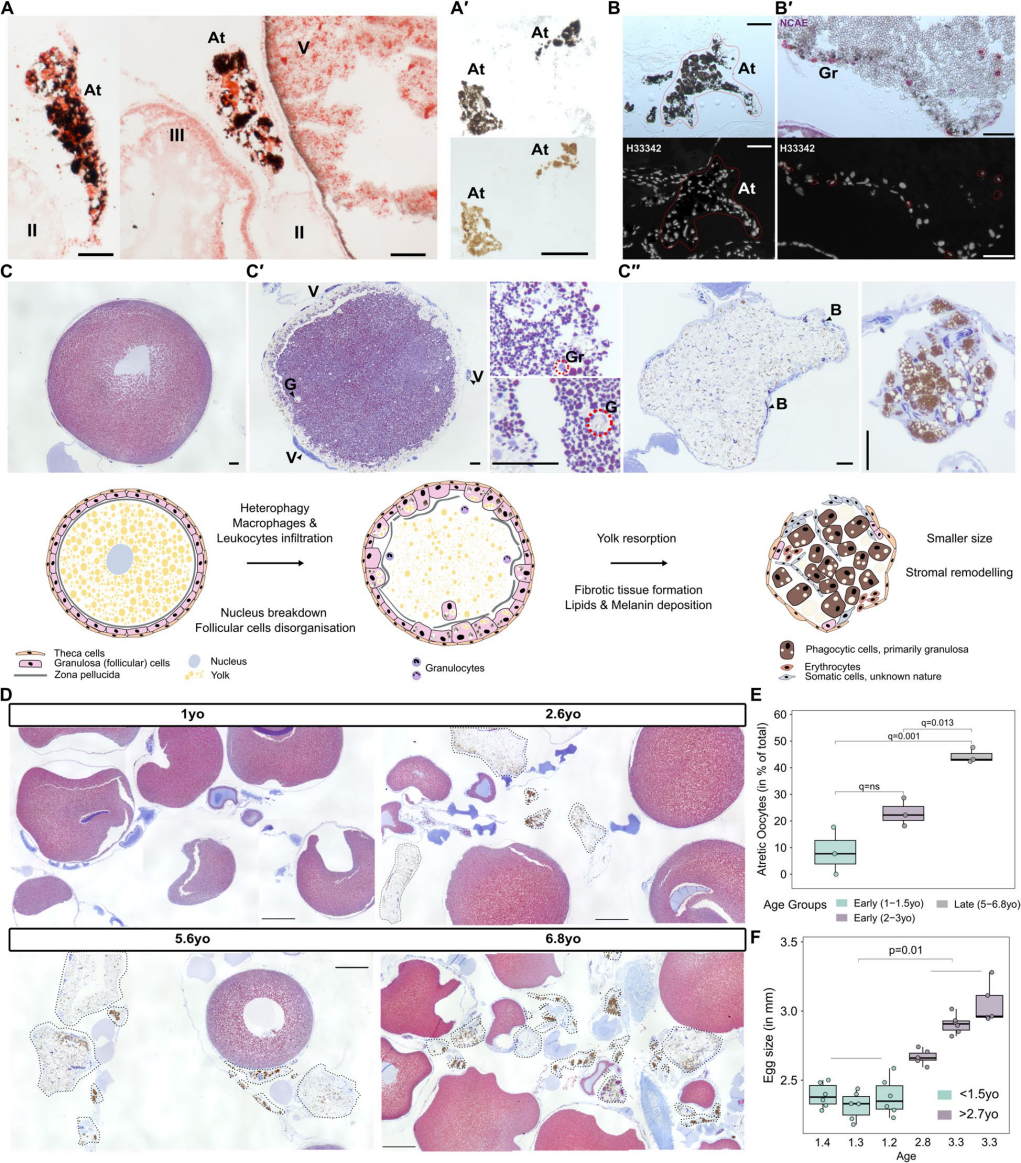
Atretic follicles accumulate melanin and lipids and are more frequent in late life females. **A** Oil red-stained sections of ovarian tissue highlighting atretic follicle. Oocyte stages are denoted using Roman numerals. **A′** Partially melanin-bleached section of an atretic follicle, showing that atretic structures are melanin-laden. **B** Advanced stage pigmented atretic structure. Hoechst 33,342 nuclei staining suggests atretic follicles are cell-laden. **B′** Early-stage atretic structure with granulocytes infiltration detected with naphthol AS-D chloroacetate esterase (NCAE) staining. **C** Mallory trichrome histological series (above) and schematic representation (below) of atresia, highlighting key molecular and morphological changes. Scale bar in **A**, **B**, **C** is 100 μm. Schematic based on and expanded from [32]. **D** Mallory trichrome-stained ovary sections across axolotl lifespan. The age is indicated above the corresponding microscopy image. Black dashed segments depict atretic follicles, highlighting their accumulation with age. Scale bar: 500 μm. **E** Quantification of atretic follicles proportion (*y*-axis) in comparison to the total number of follicles detected in histological sections of early-life axolotls (1 to 1.5 year old and 2 to 3 years old) and late life (5 to 6.8 years old). Each box corresponds to a single age group, which is also indicated with a colour. Each dot corresponds to one animal. For each animal, three to four histological sections were quantified except for 1- and 2.6-year-old animals (*n* = 2); median was used for analysis. Statistical significance between early-life groups and late-life group was evaluated by ANOVA with Tukey multiple comparisons test of means; *q*-values are indicated. **F** Measurements of axolotl egg size. *y*-axis — diameter length in mm, *x*-axis — age groups. Each box corresponds to a single animal (*n* = 3 per age group); each dot represents a single egg diameter measurement (*n* ≥ 5). Statistical significance between age groups (< 1.5 years old and > 2.7 years old) was evaluated by two-way mixed ANOVA; *p*-value is indicated. Atretic follicle (At), granulocytes (Gr), granulosa cells (G), vessels penetrating theca cells layer (V), blood cells (B)

Further histochemical assessment of pigmented ovarian structures revealed the presence of lipid content, notably higher than in nearby late-stage oocytes (Fig. 4A). Partial bleaching with 3% H_2_O_2_ and the absence of detectable autofluorescence suggested the presence of melanin as a main pigment component (Fig. 4A**′**). Additionally, the close association of these structures with oocytes and surrounding blood vessels (Fig. 3D**′**) supports their identification as atretic follicles. These follicles, irregular in shape—a hallmark of late-stage atresia—contained nucleated cells based on Hoechst staining (Fig. 4B). Additional support for their atretic nature comes from the observation of early-stage degeneration concomitant with oocyte disintegration and granulocytes’ invasion (Fig. 4B**′**).

Atresia, a progressive process of follicle degeneration and resorption [25, 26], has not been well characterised in salamander species [27]. Based on our analysis, we generated a microscopic and graphical overview of the main changes in oocytes and associated outer follicular cells during axolotl atresia (Fig. 4C, C**′′**). The process begins with oocyte shrinkage, accompanied by nuclear condensation and fragmentation of the zona pellucida (Fig. 4C, C**′**). The surrounding granulosa (follicular) cells undergo hypertrophy, while the outer thecal layer initially remains intact, providing structural support for the degenerating oocyte, and gets highly vascularised. As atresia progresses, granulosa cells proliferate and adopt phagocytic activity, ingesting and digesting yolk and, together with infiltrating immune cells, invade the oocyte cytoplasm. The oocyte becomes filled with cells, including erythrocytes, while the yolk undergoes liquefaction (Fig. 4C**′**), as previously reported in [26, 28].

In the late stage of atresia, phagocytic cells accumulate dark pigment—primarily melanin—which is not stained with Mallory trichrome staining (cells are depicted in brown in the schematic, Fig. 4C**″**). The subsequent folding of the thecal layer contributes to the irregular shape of these structures. Previous work suggests that connective tissue cells may contribute to stromal remodelling during the late stages of atresia in fish and reptiles [29, 30]. We observed the cavity of the atretic oocyte filled with somatic cells (Fig. 4C**″**); however, their precise identity is not confirmed in the present study. It was previously proposed that the yolk gets partially degraded into free amino acids [31]. Oil Red staining (Fig. 4A) revealed that lipid components of the yolk are not fully resorbed and persist in late-stage axolotl atresia. Lipid droplets are present within phagocytic cells, as a result of engulfed oocyte material, and may also persist as residual droplets in the ooplasm of atretic oocytes (Fig. 4C**″**). Additionally, pigmented atretic remnants were found close to the vasculature surface, suggesting that the digested yolk may re-enter the circulatory pool and get recycled (Fig. 3D′, Additional file 1: Fig. S3C).

Consistent with the whole-mount ovary observations (Fig. 3D), histological sections of axolotl ovaries also revealed a marked increase in atretic follicles with advancing age (Fig. 4D). The incidence of atresia was significantly higher in late-life individuals compared to early life (Fig. 4E). This age-associated rise in follicular atresia may be linked to the absence of mating in selected older females (except for one female), suggesting that the lack of reproductive activity could trigger follicular degeneration. Alternatively, the proportion of late-stage vitellogenic oocytes undergoing atresia is substantially higher, as previously suggested [32, 33]. Since older axolotls have larger eggs compared to younger individuals (based on egg diameter, Fig. 4F, Additional file 2: Fig. S4), the resorption of larger mature follicles may take longer simply due to the increased volume and yolk content.

Collectively, these results show, first, that ovarian tissue in axolotl undergoes age-related changes, including a reduction in early-stage oocytes and an increased rate of atresia. Second, despite these changes, early-stage oocytes still comprise a substantial proportion of the ovarian reserve (~ 72%) in older axolotls, indicating a preserved capacity for maturation and reproductive potential.

### Female reproductive potential persists through the lifespan

**Fig. 5:**
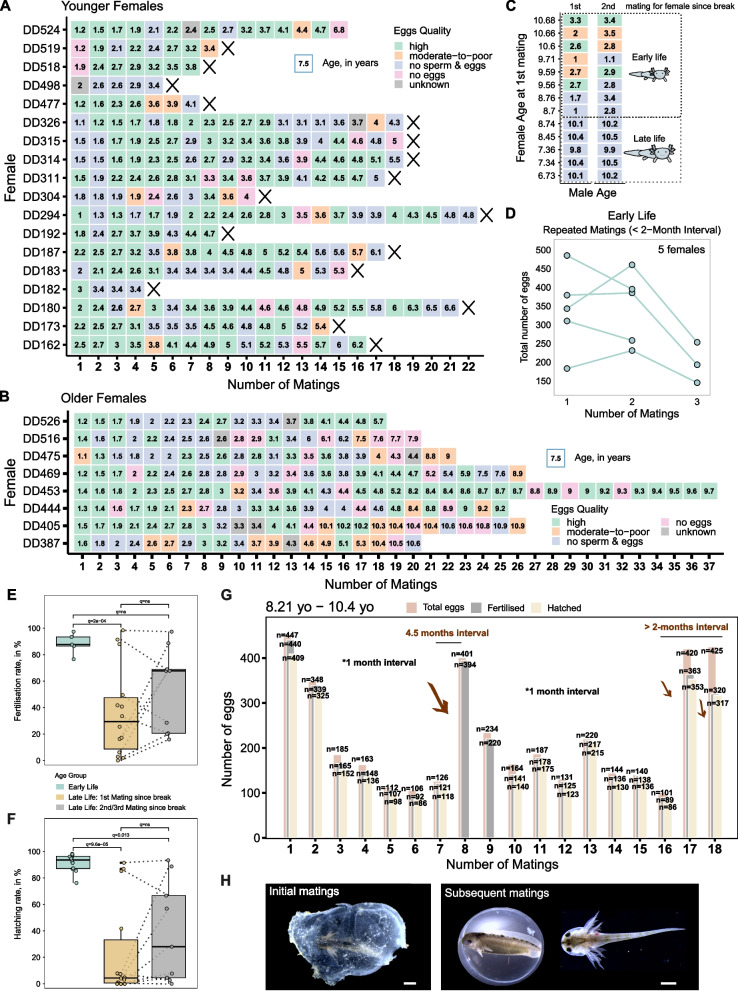
Female reproductive potential persists through lifespan. **A**, **B** Mating trends across representative females. **A** Younger females, no mating in late life. **B** Older females, mated in late life. Each box corresponds to a single mating. Number in the box denotes female age (in years) at the time of the mating, and *x*-axis represents mating number. The cross after the last mating indicates the death of the animal and therefore the end of its reproductive activity. **C** Regular mating outcome (first and second mating following a prolonged period during which female was not used) in late-life females with early and late-life males. Each box corresponds to a single mating. Number in the box denotes male age (in years) at the time of the mating, and *y*-axis represents female age at the time of first mating. Colour in **A**, **B**, and **C** indicates the quality of eggs. **D** Egg number declines (*y*-axis) in early-life females with short mating intervals. Lines connect data points from the same female; *x*-axis represents mating number. **E**, **F** Fertilisation. **E** larval hatching and **F** rates per mating in early-life and late-life females during the first and second/third matings following a prolonged period during which female was not used. Colours indicate age group. Dotted lines connect data points from the same female. In **E**, *n* of matings in early life = 5, in late life (first mating) = 14, and in late life (second or third mating) = 9. In **F**, *n* of matings in early life = 12, in late life (first mating) = 14, and in late life (second or third mating) = 9. Statistical significance between age groups in **E** and **F** was evaluated by Kruskal–Wallis test and pairwise Dunn’s tests with Bonferroni correction, and *q*-values are indicated. **G** Representative mating history of late-life female. The age of female at the time of first and last mating is depicted in the top left corner of the plot. Bars correspond to number of eggs produced and embryos viability, and *x*-axis represents mating number. The average interval between matings is approx. 1 month, except between matings 7 and 8, 16–18. *n* denotes the number of eggs/embryos. **H**. Representation of axolotl larvae and jelly quality from females in late life: above, first matings following a prolonged period during which female was not used, below, after a few subsequent matings. Scale bar: 1 mm

The ability of axolotls to regenerate ovarian tissue [17], along with our finding that the ovarian pool is preserved even at advanced age, prompted us to further investigate fertility in late-life females. Previously, we pointed to an age-related increase in no-egg matings (Fig. 2C, D**′**). Thus, we analysed individual mating histories (Fig. 5A) to determine whether early-life females exhibited a consistent, age-associated reproductive decline across individuals or whether inter-animal variability had a greater influence. The occurrence of no-egg matings was not strongly correlated with age and often resulted from a lack of sperm deposited, possibly due to failed courtship interactions (Fig. 5A). Often, these females were not mated in late life as they were usually retired or sacrificed around 4–5 years of age, when fertility was thought to decline, resulting in fewer older females remaining alive (Fig. 5A, Additional file 2: Fig. S5).

Similar observations of no sperm deposition during pairings were made in females mated in late life (Fig. 5B). Using a multinomial logistic regression model, we examined how age influences the likelihood of mating outcomes in both females and males, with a focus on the event of sperm deposition failure (Additional file 2: Fig. S6). Essentially, we attempted to predict the outcome pairing females and males of particular age. However, the model performed poorly (Hosmer–Lemeshow test *p* = 0.005, pseudo-R^2^ = 0.012; Additional file 2: Fig. S6A). The age parameter was not discriminative enough to accurately predict the mating success, and events such as ‘no eggs’ and ‘no sperm and eggs’ were misestimated at older ages (Additional file 2: Fig. S6A′). Despite the weak fit, the analysis still showed age-related patterns in mating outcomes (Additional file 2: Fig. S6A, B). An increasing male age was associated with a higher likelihood of sperm deposition failure, which subsequently results in a no-egg event. Z-scores, used here as a metric of each sex contribution to mating outcomes, highlighted the strength of this association (*Z* = 6.2, *p* = 5.9e-10 vs. female age: *Z* = − 0.86, *p* = 0.38; Additional file 2: Fig. S6B). Conversely, female sex carries weight in ‘no egg deposition’ event (*Z* = 3.62, *p* = 0.0003 vs. male age: *Z* = 1.46, *p* = 0.14). The chi-squared test (Additional file 2: Fig. S6C) further confirmed the association between age and mating outcomes for both females (*χ*^2^ = 33.1, *p* = 9.6e-06) and males (*χ*^2^ = 96, *p* < 2.2e-16). This suggests that age alone exerts a rather modest effect on expected reproductive outcomes, and that the impact of male age is more pronounced compared to female age. In particular, males may exhibit stronger behavioural unresponsiveness with age, leading to reduced mating success.

We further investigated the impact of male age in unsuccessful matings by pairing early- and late-life males with late-life females (Fig. 5C). We observed that the likelihood of sperm deposition and successful first and second matings was moderately higher when males younger than 4 years were used, regardless of female mating history (Fig. 5C). These results suggest that courtship interactions may become less effective as males age.

Individual mating histories also revealed an increased rate of moderate-to-poor-quality eggs in females above 4.5 years old which may result from frequent matings. In particular, younger females have at least a 2-month breaks between matings, while older females are usually kept in shared mating tanks for a prolonged time. This results in the increased frequency (approx. 1-month intervals). This shorter recovery time may limit oocyte regeneration, reducing egg numbers which, despite being fertilised and viable, are still assigned to moderate to poor. This is further shown on early-life females (Fig. 5D). With increased frequency of matings, females lay fewer eggs. Additionally, after a few years interval, females initially produce moderate-to-poor-quality eggs (high number but poor quality and low number of hatched larvae).

To further challenge this notion, we closely analysed matings involving 6.5–10.5 years old females to evaluate the viability and health of their offspring (Fig. 5E, F, G, H, Additional file 2: Fig. S7). Most females initially had a low proportion of fertilised eggs (median around 30% of the total, Fig. 5E) and hatched larvae (ranging from 0 to 92% of the total, Fig. 5F). Additionally, the quality of jelly was lower than that in early-life females (Fig. 5H, left). However, even at the edge of their lifespan and despite high-frequency matings, axolotls laid developmentally competent eggs (Fig. 5E, F, G), with females consistently exhibiting high reproductive rates.

The low hatching rate observed in late-life females upon first mating after a long gap may be attributed to alterations in jelly quality. After prolonged periods without use, the jelly tends to harden and lose its uniform structure, exhibiting reduced turgidity (Fig. 5H, left). The observed changes could result in reduced protection so that eggs are more vulnerable to mechanical stress or disrupt gas exchange. As a result, fertilised eggs may develop into embryos with defects, affecting hatching rate.

Yet, if females are allowed to recover, the number of eggs produced would increase (Fig. 5G). Moreover, the quality of jelly, proportion of fertilised eggs, and number of hatched larvae can improve in subsequent matings (Fig. 5G, H, Additional file 2: Fig. S7). Taken together, these data suggest that axolotls retain fertility even in old age.

## Discussion

In this project, we investigated reproductive rates in axolotls and explored the impact of ageing on fertility, focusing on key factors such as mating history statistics, ovarian tissue analysis, and the breeding potential of older individuals. We provide evidence that axolotl reproductive ability is retained into old age, within the average lifespan of these species, and that reproductive declines can be partially reversed through regular breeding. The limited reproductive senescence highlighted in this study supports the notion that the axolotl may exhibit negligible senescence.

Axolotls are capable of extreme regeneration, restoring complex structures. However, beyond the extensive regenerative capacity, they display limited signs of physiological change with age and extreme cancer resistance [16]. It has been proposed that axolotls show signs of fertility loss close to 4–5 years of age (anecdotal observations [18]). In light of the lack of studies on this phenomenon, we sought to further investigate the reproductive potential of axolotls throughout lifespan.

Amphibians require specific environment and conditions (temperature, humidity, photoperiod) to trigger mating, but systemic data on mating success from colonies, such as historical records, are rarely reported [21, 34]. This study offers a significant contribution to the topic by analysing records spanning more than a 15-year history of our axolotl colony. We reveal that axolotls can reach mating success rates of around 50% in captive breeding. This aligns with comparative studies on anurans [21, 35, 36]. Interestingly, the breeding success rate showed only minor variation throughout the year, contrary to the widespread notion that mating rates decrease in summer months. However, the colony records exhibit heterogeneity, with some individuals engaging in mating at lower rates than others, a pattern repeatedly observed in females paired with older males and in cases of incompatibility between particular young male and female breeders. This heterogeneity could negatively affect mating statistics and artificially introduce lower mating success rates. Still, as a reproductive strategy, mating and fertilisation success vary naturally among individuals; analysing all matings likely offers a more realistic view of reproductive dynamics.

Furthermore, we hereby report egg classification metrics which indicate that high-quality eggs are the predominant outcome of the matings, suggesting a high potential for successful hatching and larval development. However, we also observed that transgenic animals (excluding knockout lines) exhibit a slightly lower rate of successful matings along with a decreased proportion of high-quality eggs. This difference in reproductive rates could be attributed to genome challenges inherent to the transgenesis method. In particular, I-SceI- or Tol2-mediated transgenesis may result in multiple DNA insertion sites across the genome, while CRISPR/Cas9 implementation may also lead to off-target integrations [22, 37]. Such events may interfere with genes related to fertility or gametogenesis [38]. Alternatively, if few founders are available during line generation, inbred or closely related pairs might be used, increasing the likelihood of repeated unsuccessful matings or affecting the quality of eggs.

Fertilisation and hatching rates in amphibians may also vary, but they are relatively high for species for which reliable husbandry has been developed. Representative examples include *Xenopus* species [21, 35, 36] or newts [39, 40]. According to our colony records, the fertilisation rate in WT or DD axolotls is close to 80% in the event of successful mating. The observed clutch size has a median of 350 eggs in early-life individuals, decreasing to around 239 eggs in late life. This reduction may be attributable to several factors. One possibility is the existence of age-related reproductive decline. However, it could also be influenced by alternative factors such as body size. Axolotls exhibit indeterminate growth [18, 41]; therefore, older individuals are predictably bigger and can accommodate a larger clutch of eggs. Consistent with this possibility, we hereby show that older axolotls lay larger eggs, corroborating previous observations [39, 42]. Assuming similar resource availability, if older axolotls invest more in offspring size rather than number [43], late-life females are expected to produce fewer but larger eggs compared to early-life females.

We also analysed the contribution of males to the reproductive outcome. In general, male axolotls demonstrated high fertilisation ability, in a few cases even above 8 years of age, and could be used repeatedly for mating at 2-week intervals. At the same time, we found that the absence of eggs was more likely to result from no spermatophore deposition by males, increasing with age. The multinomial logistic regression model and chi-square test indicate that increasing male age best predicts the lack of sperm deposition, but male age is less predictive of no-egg events than female age. We also observed that the mating success rate in late-life females was moderately higher when younger males are used. With that, we propose that male physical potency, including sperm availability, is largely maintained across the lifespan. This is similar to findings in the Mississippi gopher frog, where sperm quality remained high in older individuals [44], and in the sword-tailed newt, where males retained fertility at the age of 34 [45]. However, behavioural changes may influence male reproductive outcomes. As axolotls get older, they appear to become less active [46]. These could interfere with essential reproductive behaviours (the courtship dance) leading to unsuccessful mating attempts despite maintained physical capability. Yet, further testing is required, including the assessment of reproductive hormone levels, sperm motility, and behavioural studies.

We further observed sex-specific differences in contributions to mating outcomes. In particular, late-life females were associated with lower egg quality. In the absence of a prolonged gap in reproductive activity, oocytes may be exposed to the accumulation of damage over time. If turnover is constrained when reproduction does not occur, oocytes may persist in the ovary. This prolonged retention can increase susceptibility to oxidative stress and DNA damage [8, 47, 48], potentially compromising egg quality and reproductive success since mating break. However, once females are regularly mated, the quality of oocytes, after reproduction-induced regeneration [17], may improve. This is supported by our observations of late-life breeding females, in which egg quality and larval hatching improve with repeated matings. Further experimental work is needed to determine the existence of molecular and cellular damage in oocytes after prolonged periods of reproductive inactivity.

In this study, we also observed that axolotls preserve their ovarian pool throughout lifespan, enabling breeding events. Yet, we also observed the existence of time-related changes, as evidenced by an expansion of the connective tissue and a reduction in early-stage oocytes. This led us to obtain insights into follicular atresia in salamanders. This is the process of the natural remodelling of the ovary, the extent and cause of which can vary among species and individuals [26, 29, 49]. The increased rate of follicle breakdown in late-life axolotls may be influenced by factors such as reproductive history, environmental conditions, or ageing. As proposed above, if animals are not mated, the need to maintain a large follicle pool decreases; thus, vitellogenic oocytes may be eliminated. An increased clearance burden may be introduced by larger atretic follicles in older individuals, as their resorption requires more time due to their greater volume, potentially leading to prolonged persistence within the ovary. Although atresia can also be induced by stress factors, this possibility is unlikely, as all animals were maintained under similar conditions.

The incidence of atresia may be associated with age, suggesting animals exhibit slow but progressive reproductive deterioration. However, it may also result from a reduction in investment in egg production. Atresia could enable the reallocation of resources; for example, during periods of starvation, yolk-containing oocytes are reabsorbed first [25, 26]. In support of that, we observed that atretic remnants were found close to the vasculature. Studies in teleosts demonstrate that atretic follicles upregulate *apoa1* and *apoc1*, with hypertrophied follicular cells at least overexpressing *apoc1* [28]. ApoC-I and ApoA-I are required for high-density lipoproteins (HDL) maturation and activation of the reverse cholesterol transport pathway, suggesting a role of follicular cells in lipid transport. In line with this, yolk proteins were detected in bloodstream being associated with HDLs [28, 50]. Alternatively, an increased rate of atresia could result from a shift from high egg numbers towards fewer yet larger eggs, as seen as axolotls get older, with atresia providing raw materials enabling egg size expansion. Further research should examine these possibilities further.

The aforementioned considerations support the existence of an overall stabilisation of the reproductive output in mature axolotls. Specifically, declines in the number of early-stage oocytes, the total egg numbers, and a shift towards a lower proportion of high-quality eggs or an increase in no-egg events are prone to plateau around 4 years of age. This coincides with the onset of less active behavioural patterns in axolotls. The initial decline in reproductive output followed by stabilisation may reflect an adaptive strategy to support longer survival [43, 51]. By reducing energy investment in offspring, more resources become available for somatic maintenance. Despite this reduction, reproductive fitness is retained, as evidenced by successful breeding in late-life females. Remarkably, females aged between 6 and nearly 11 years are able to produce hundreds of eggs. While the initial batches had a proportion of embryos with malformations or high numbers of unfertilised eggs, further batches could yield healthier offspring. These findings lay the groundwork for exploring the broader use of late-life axolotls in mating, offering a dual benefit of increased productivity and improved animal welfare through extended use.

## Conclusions

Through a lifespan-wide assessment of fertility in axolotls, we report that their reproductive potential peaks after sexual maturation. Clutch size, egg quality, and mating success rate are the highest at approximately 2 years of age. These reproductive traits gradually decline until 4 years of age, after which they plateau, exhibiting limited changes. Importantly, we demonstrate that axolotls retain early-stage oocytes into advanced age and provide an in-depth characterisation of follicular atresia in this salamander species. Our findings reveal that axolotls remain fertile throughout their expected lifespan, exhibiting limited reproductive senescence.

### Limitations

In this study, we used axolotl colony-history records. Although we expanded these records by conducting arranged matings in both early- and late-life animals, they still predominantly consist of breeding records that were not experimentally designed to test fertility. We acknowledge that these records may be biased. Indeed, some individuals are particularly unsuccessful breeders, which can introduce negative bias into mating statistics. However, because mating and fertilisation naturally vary between individuals, including all matings likely provides a more accurate picture of reproductive dynamics.

Although we introduce the idea that behavioural changes in males with age may be among the primary causes of the increased incidence of no-mating events, we have neither provided reproductive behaviour metrics nor conducted behavioural studies on axolotls. These topics, along with further physiological assessments of males, including sperm quality and motility and their hormonal changes, should be addressed in future work.

We also assessed the oocyte pool using whole-mount ovarian samples. While this method provides a broad overview of oocyte distribution, it does not allow for the detection of Stage 0 oocytes. These earliest-stage oocytes can only be reliably identified through histological analysis. Additionally, whole-mount imaging does not allow for fully accurate quantification of early-stage oocytes due to structural overlap and limited resolution. Some margin of error is expected in these counts. As a result, our analysis may underestimate the oocyte reserve.

Although we observed a large number of matings in early-life animals, the dataset on the reproductive history of late-life females was comparatively limited. Within the axolotl facility, females were typically retired from breeding or sacrificed at around 4–5 years of age. As a result, fewer older females were available for breeding. While statistical analyses partially addressed this constraint, it highlights the need to expand the dataset for a more detailed characterisation of axolotl reproductive health across ageing.

## Methods

### Ethics and animal husbandry

Animal handling was performed in compliance with the current German Animal Welfare Act and legislation from the state of Saxony. Axolotls (*Ambystoma mexicanum*) were bred and maintained at the CRTD facility (Dresden, Germany). Axolotls were housed in individual aquaria at ∼18–20 °C and a 12 h/12 h light/dark cycle.

Tissue collection was authorised under the licence TVT 5/2021 issued by the animal welfare authorities.

### Animal mating

Male and female axolotls were transferred to mating tanks and maintained at a reduced temperature (15–16 °C). Plastic leaves were placed in the tanks to provide a suitable surface for egg deposition by the female. The animals remained in the mating tanks for up to 5 days.

The male would initiate courtship by nudging the female with his snout, followed by the deposition of spermatophores in the tank. The female would then follow the male and pick up the spermatophores with her cloaca for egg fertilisation [52]. When the female started egg deposition, or after a maximum of 4 days, the male was returned to his individual holding. Often, the female began laying eggs approximately 18–24 h after spermatophores deposition. Early-life (≤ 4.5 years old) females were mated at intervals of at least 2 months.

Matings of late-life (> 4.5 years old) females and late-life males were primarily conducted in group settings, with either two males and one female or two females and one male housed together in a single mating tank for an extended period. Under these conditions, mating could occur at intervals of approximately 1 month.

In particular settings, matings of a late-life female and an early- or late-life male were performed under regular conditions. The axolotls were transferred to a mating tank and kept for up to 5 days. Afterwards, they were returned to their individual holding.

### Egg collection and quantification

Following mating, axolotl eggs were collected and transferred to a separate tank. Within 24 to 48 h, the eggs were inspected to identify and/or remove infertile or damaged ones. The eggs were maintained at room temperature (RT) and monitored until hatching, which regularly occurs within 2 weeks.

For a subset of matings where the number of eggs was reported, only the total number of eggs was quantified. To compare fertility between early-life and late-life females, total number of eggs, number of fertilised eggs, number of embryos with developmental defects, and total number of hatched larvae were recorded.

### Egg quality

Eggs were classified based on viability, morphology, jelly layer characteristics, and quantity per mating and defined as follows:

- High-quality: More than 60–70% of eggs were viable and fertile, morphologically normal with a clear outer jelly layer, and yielded viable embryos. Egg counts were typically ≥ 200 per mating.
- Moderate-to-poor quality: Fewer than 50% of eggs were viable and fertile. This included either morphologically normal eggs with a clear jelly layer but in low numbers (< 150 eggs per mating) or morphologically abnormal, deformed eggs with reduced developmental potential and a loose, turbid, sensitive jelly layer, occurring in high (≥ 200 eggs per mating) or low numbers.

### Egg fertilisation and larvae hatching rates

Egg fertilisation rate was calculated 2–3 days post-mating, and larvae hatching rate was calculated in 13–15 days post mating, as the proportion (in percent) of total eggs laid. First mating in late-life females since long break implies that females were mated for the first time since the several years of no breeding (on average, 4 years without use). In cases where repeated matings for a single female were possible, fertilisation and hatching rates were also reported after the subsequent second or third mating. The sample sizes were as follows:

- In fertilisation rate analysis, number of matings in early life = 5, in late life (first mating) = 14, and in late life (second or third mating) = 9
- In hatching rate analysis, number of matings in early life = 12, in late life (first mating) = 14, and in late life (second or third mating) = 9

### Axolotl colony records

Reproductive records were collected in the axolotl colony first established at the Max Planck Institute of Molecular Cell Biology and Genetics, Dresden (2000–2012) and later transferred to the Center for Regenerative Therapies Dresden, Germany (2012–now). The presented analysis on mating statistics from the colony was based on records from 2010 to September 2024 (with occasional matings in 2025), since the egg quality classification used in this study has been applied for evaluation since 2010 and maintained by the same animal caretakers throughout this period. However, due to limited data on post-mating egg numbers, this study period for the number metric was expanded to include records from 2001 to 2025, covering a span of 25 years and incorporating records by different animal caretakers. Accordingly, all data statistics and analyses were based on records up to May 2025, whereas data on egg numbers per mating were based on records up to August 2025 (Additional file 3: Table S1 and Additional file 4: Table S2).

Mating records included the male and female used for each mating, the date animals were placed in the mating tank, dates of spermatophore and egg deposition (if this took place or not), and return dates to individual holdings. Age at the time of mating was calculated if the date of birth information was known.

Matings that involved at least one knockout (KO) animal or if resulted from in vitro fertilisation (IVF) events were excluded from the analysis. For the transgenic versus DD/WT mating analysis, we defined matings as transgenic if they involved either a transgenic female or both animals were transgenic. Matings involving a WT/DD female and a transgenic male were excluded from the transgenic group, based on the assumption that the male transgenic status has a lesser influence on egg quality and number compared to the maternal contribution.

Mating outcomes were classified as follows:

1. Successful — Both spermatophores and eggs were deposited. When spermatophores are not visible but eggs have been deposited, it was inferred that spermatophores have been deposited.
2. Only sperm — Only spermatophores were deposited, but no eggs.
3. Unsuccessful — Neither spermatophores nor eggs were deposited.

### Data visualisation

All datasets analysis and visualisation were performed in R (v4.3.2). In bar plots, we reported the proportions (in percentages) of the parameter per age group or category. Unless stated otherwise in the figure legend, the total sample size per category or age group was reported.

Egg number distributions between early- and late-life females were visualised with the density plot, a kernel density estimate, which provides a smoothed version of a histogram (geom_density, ggplot2 v3.5.0). Colour palette was adapted from the package RColorBrewer (v1.1.3).

All schematics and drawings were created in Inkscape (v1.3.2).

### Chi-square test

To investigate the relationship between increasing age and egg quality, and age and mating outcome, we applied a chi-squared test of independence using the chisq.test() R function. This statistical test is appropriate for evaluating associations between categorical variables. The dataset was stratified by age and sex, and only individuals with known age data were included in the analysis. Age was treated as a categorical variable (as factor), and for egg quality analysis, it was stratified into the following groups (in years): 1.2–2, 2–3, 3–4, 4–4.5, and > 4.5. Egg quality was classified into three categories: high quality (in females *n* = 1774, in males *n* = 1669), moderate-to-poor quality (in females *n* = 318, in males *n* = 305), and no eggs (in females *n* = 1594, in males *n* = 1691).

For mating outcome analysis, age was stratified into the following groups (in years): 0.8–2, 2–3, 3–4, and > 4. Mating outcome was classified into three categories: eggs (in females *n* = 2172, in males *n* = 2124), no eggs (in females *n* = 449, in males *n* = 503), and no sperm and no eggs (in females *n* = 1186, in males *n* = 1294).

We report the test statistic (*χ*^2^), associated *p*-value, and standardised residuals. For data visualisation, we used the corrplot package (v0.94), with the visualisation method set to ‘square’ to display the standardised residuals.

### Multinomial logistic regression model

A multinomial logistic regression model was employed to evaluate the effect of axolotl female and male age (independent variable, predictor, numerical) on the likelihood of different mating outcomes, classified into three categorical levels: eggs (both spermatophores and eggs were deposited), no eggs, and no sperm and eggs. Only mating events for which age data in both females and males were available were included in the analysis. We used the multinom() R function from the nnet package (v7.3.19).

The sample size was as follows: eggs (*n* = 1420), no eggs (*n* = 277), and no sperm and eggs (*n* = 657). To address an uneven distribution of outcome categories, we explored downsampling (reducing ‘eggs’ category) and assigning class weights inversely proportional to the frequency. These adjustments yielded similar model performance results. Therefore, the original data was used for consistency.

The model estimated the relative log-odds (coefficient) of each outcome category relative to a chosen reference category. The ‘eggs’ category was selected as the reference for two primary reasons: (1) To assess how increasing age affects the likelihood of no-egg or failed sperm deposition event relative to producing eggs, and (2) it represents the most frequent category in the dataset, providing a statistically stable baseline for comparison.

Model fit was assessed by the Hosmer–Lemeshow test to evaluate if the observed and expected values differ and by computing McFadden pseudo-*R*^2^. We used the logitgof() R function from the generalhoslem package (v1.3.4) and pR2() — from the pscl package (v1.5.9).

multinom() function does not supply *p*-value; therefore, we computed *p*-values using *Z*-tests. Z-scores were derived as follows: Z-score = coefficients/standard errors. *p*-values were estimated from a two-tailed *Z*-test: *p* = 2 × (1 − pnorm(abs(Z-score))).

To extract the estimated probability of each category from the model, we used nnet::predict() function via predict(model, type = "probs"). Further, the probabilities were visualised by female and male age in ggplot2 using geom_point().

To further assess the accuracy of the model, predicted and observed outcomes were compared. For that, observed outcomes were assigned binary values, where 1 indicated that the outcome was present and 0 was absent. Predicted probabilities (in 0 to 1 range) were extracted from the model output for the same categories. Observed and predicted data were grouped by sex and age groups (in years): 0.8–3, 3–6, 6–9, and > 9. For each sex and age group, the mean proportion of each outcome was calculated separately for the observed and predicted values.

### Ovary collection

For tissue collection, axolotls were sacrificed by 0.1% benzocaine (Sigma) overdose. Ovaries were collected by dissection, washed in A-PBS (70% PBS), and primary fixed in 4% paraformaldehyde (PFA) in 100-mM phosphate buffer for 2 h at RT and then were transferred to 4 °C.

Fixed samples for Oil Red O and naphthol AS-D chloroacetate esterase (NCAE) staining were washed in A-PBS and embedded in Epredia™ Cryochrome media. The samples were sectioned in a cryostat at 5–10 μm. Sections were collected in Epredia™ Superfrost slides, dried for 1 h at RT, and stored at − 20 °C.

Fixed samples for the morphological analysis with Mallory trichrome staining were embedded in Technovit T7100 [53]. For this, the fixed ovaries were dissected into smaller pieces; some were postfixed with 1% glutaraldehyde in 100-mM phosphate buffer and dehydrated in a graded series of ethanol/water (30%, 50%, 70%, 90%, 96%, 15 min each) followed by three changes (20 min each) in 100% ethanol on molecular sieve. The samples were infiltrated stepwise in Technovit 7100 methacrylate resin infiltration liquid (including hardener 1) using the following steps: 25%, 50%, and 75% resin in 100% ethanol for 1 h each, pure resin for 2 h, and pure resin overnight. Ovaries were finally embedded and cured by adding hardener 2. The resin blocks were stored at 4 °C and sectioned in a rotary microtome at 2 μm. Sections were collected in Epredia™ Superfrost slides, dried for 1 h at RT, and later stored at RT.

### Oocyte developmental staging

Axolotl ovary tissue across ages that were stored in 4% PFA at 4 °C was used for the imaging and quantification. The animal number and ages were as follows: 1.3 years old (*n* = 2), 1.8 years old (*n* = 3), 3 years old (*n* = 3) — early life group, 4.4 years old (*n* = 1), and 6.8 years old (*n* = 3) — late-life group, and six samples per animal were quantified. Animals aged 3 and 4.4 years were previously used for breeding, whereas the other animals had not been mated before.

Oogenesis is persistent in the adult ovary, and oocytes at various developmental stages are distributed throughout the ovarian tissue without being confined to a specific location [17]. Therefore, samples were collected along the rostro/caudal axis of the ovary and not at the particular anatomical location. Sample sections, obtained for the analysis, included both the peripheral tissue containing early-stage oocytes and the deeper layer of later-stage oocytes, as illustrated in Fig. S2.

Whole-mount ovary samples were used to identify the proportion of early- and late-stage oocytes. Oocyte staging was based on structural characteristics, including yolk accumulation, pigmentation, and size ratios. Adapted staging was based on schemes in axolotl [17] and *Xenopus* [54–56]. We classified oocytes into early stages (I–III) and late stages (IV–VI), calculated the proportion of early-stage oocytes relative to the total number in each sample, and reported the values per animal. To ensure consistency across samples, atretic follicles were also included in the quantification, as they represent follicles undergoing degenerative processes. Only atretic follicles with a well-defined round or oval structure were included, while dispersed remnants were excluded due to the difficulty in determining whether they represent one or multiple follicles.

This approach provides an overview of whether the distribution of oocyte developmental stages and the reproductive potential of the ovaries are preserved during ageing. Stage 0 oocytes (oogonia to the diplotene) were excluded from this analysis because whole-mount imaging lacks the spatial resolution necessary to distinguish and accurately quantify these early developmental stages.

### Mallory trichrome staining

Slides were baked at 60 °C for 2–3 h prior to staining. For staining, slides were incubated for 15 min at 60 °C in a 1:1 mixture of 3% potassium dichromate and 10% hydrochloric acid prepared in 95% ethanol. Slides were then rinsed under running tap water for 10 min and dried at 60 °C.

Staining was performed using a Mallory stain solution containing 0.5% phosphotungstic acid, 1% orange G, 0.5% aniline blue, and 1.5% acid fuchsin. Slides were incubated at 60 °C for 30 min. After staining, slides were sequentially dipped in distilled water, followed by 40%, 70%, 96%, and two changes of 100% ethanol. Finally, the slides were air-dried, mounted with Entellan, and covered with coverslips.

### Naphthol AS-D chloroacetate esterase staining

Granulocytes in ovarian cryosections were detected with the naphthol AS-D chloroacetate esterase (NCAE) kit (Sigma-Aldrich) following manufacturer’s protocol with several adjustments. Prior to staining, slides were rehydrated in A-PBS for 5 min at RT. Slides were further incubated in the staining solution in a water bath at 37 °C for 15 min (covered from light). Later, slides were rinsed in water for 2 min at RT and incubated in Hoechst 33,342 nuclear stain in A-PBS (1:10 000) for 10 min at RT covered from light. Slides were washed twice in A-PBS for 5 min at RT and dried and mounted with the Dako fluorescence mounting medium (Agilent Technologies, Inc.).

### Oil Red O (ORO) staining

A 0.5% stock solution of Oil Red O (Sigma-Aldrich) was prepared by dissolving 0.5 g of ORO in 100 mL of 99% (v/v) isopropyl alcohol. The working solution was prepared by mixing 1.5 parts of the ORO stock solution with 1 part of distilled water and was used within 6 h.

Cryosections were brought to RT and washed once with A-PBS for 3 min. The sections were then incubated with the ORO working solution for 5 min, with approximately 1 mL of solution applied per slide containing 4–6 sections. The solution was added gently to avoid washing out or detaching the sections. Following incubation, the sections were washed and placed in a chamber containing A-PBS for 30 min. Imaging was performed immediately after staining.

### Bleaching with hydrogen peroxide

Cryosections were brought to RT and washed once with A-PBS for 3 min. The sections were then incubated with the H_2_O_2_ (hydrogen peroxide) 3% working solution in distilled water for 30 min at RT in the dark, with approximately 50 µL applied to the area of interest. The solution was added gently to avoid washing out or detaching the sections. The sections were monitored throughout the incubation, and the solution was refreshed if drying occurred. Observations under the microscope were performed every 10 to 15 min. Depending on the thickness of the sections and the amount of pigment, the incubation was extended by an additional 20 min, for a maximum of 70 min. The sections were incubated and monitored until the dark pigment faded to a yellow colouration. After incubation, the sections were washed with distilled water by placing the slides in a chamber containing water.

### Atretic follicles visualisation and quantification

The schematic of atresia progression was adapted from [32] and significantly expanded in the present study.

For quantification, Mallory trichrome-stained sections were used. We calculated all observed follicles (stages I–VI), excluding germ cells. Then, we quantified the proportion of atretic follicles relative to the total number in each section. For each animal, at least three sections were used for quantification, except for the 1- (*n* = 2) and 2.6-year-old (*n* = 2) animals. We reported the median value per animal. Age groups (three animals per group) were as follows: 1–1.5 years old — early life, 2–3 years old — early life, 5.6–6.8 years old — late life.

In cases of clustered pigmented atretic regions separated by thin fibrotic tissue, we counted every second distinct pigmented structure as one atretic oocyte to avoid overrepresentation of fragmented debris, particularly in older samples.

### Egg size

Egg size differences between younger and older axolotls were assessed. Eggs were collected from animals younger than 1.5 years and from animals older than 2.7 years, within 8 h of post-laying. For each animal, at least 5 eggs were imaged, measuring egg diameter and excluding the jelly. The selected age ranges were chosen as females reach sexual maturity around 12 months, while the rapid animal growth phase ends near 1.7 years [41].

## Imaging

Predominantly, axolotl eggs, spermatophores, and whole-mount ovarian samples were observed and imaged under a Stereomicroscope System SZX10 (Olympus); the pictures were obtained with the Digital Microscope EP50 Camera (Olympus). Atretic follicles and eggs for egg size measurements were imaged using a Zeiss AxioZoom V.16 microscope and Zen software (Zeiss).

Imaging of Mallory Trichrome-, Oil Red O-, NCAE-, and Hoescht 33,342-stained ovary samples was conducted using a Zeiss AxioZoom V.16 microscope and Zen software (Zeiss).

Fiji (ImageJ 1.54f) was used for image analysis and quantification.

### Statistics

Statistical analysis was performed in R (v4.3.2). Prior to each analysis, datasets were tested for normality using the Shapiro–Wilk test. InFig. 2A, the difference between proportions in early- and late-life groups was calculated by proportion test; in Fig. 2B, the statistical significance of egg number distributions were calculated by Kolmogorov–Smirnov test; and in Fig. 2D and D**′**, the difference between proportions in selected age groups was calculated by proportion test with Bonferroni correction. In Fig. 2E and E**′**, the median egg number difference among age groups was calculated by Kruskal–Wallis test and pairwise Dunn’s tests with Bonferroni correction. In Fig. 3, the statistical significance between early- and late-life groups was calculated by Wilcoxon test. In Fig. 4E, the statistical significance between early-life groups and late-life group was evaluated by ANOVA with Tukey multiple comparisons test of means. In Fig. 4F, two-way mixed ANOVA was applied. In Fig. 5D and E, the statistical significance between the early-life group and late-life groups was calculated by Kruskal–Wallis test and pairwise Dunn’s tests with Bonferroni correction. For the abovementioned tests, packages stats (v4.3.2) and FSA (v0.9.5) were used.

## Supplementary Information

Additional file 1. Figures S1-S3. Fig. S1: Mating success rates remain consistent across different seasons. **A**, Axolotl colony mating statistics, shown as percentage (y-axis). n denotes the total number of matings. **B,** Representation of reproductive material: **1**, fertilised eggs, **2**, unfertilised, necrotic eggs. Arrows: yellow indicates jelly layer, white denotes axolotl embryo; **3**, spermatophore. Arrows: red indicates packet of sperm, yellow denotes conical gelatinous base. Scale bar in 500 μm. **C**, Matings distribution across years between wildtype axolotls and transgenic lines. y-axis denotes the number of matings within respective genotype groups. Fig. S2: Axolotl ovarian tissue collection. Schematic of collection area. Grey dashed lines and red area indicate how samples were collected along the rostro/caudal axis of the ovary. Fig. S3: Histological and whole-mount assessment of axolotl ovarian tissue. **A**-**B′**, Mallory trichrome stained sections of axolotl ovary: **A** and **B′** from 6.8-year-old animal, **B** from 3.1-year-old animal. **A**, germ cells (gc) based on morphological characterisation, in close proximity to Stage I oocyte. Scale bar 100 μm. **B**-**B′**, Visualisation of connective tissue deposition. Collagen (c) fibers (stain blue), nuclei (n). Scale bar in **B** 50 μm, in **B′** upper 100 μm and lower 50 μm. **C**, Pigmented atretic remnants are located in close proximity to the vasculature. Scale bar 200 μm. Atretic follicle (At), Atretic follicle remnants (At.r), vessels (V), Stage II oocyte (II).

Additional file 2. Figures S4-S7. Fig. S4: Egg size difference with age in axolotls. Axolotl eggs from 1.2-year-old (left) and 3.3-year-old (right) animals. Scale bar 500 μm. Fig. S5: Distribution of axolotl age at death, stratified by sex. The frequency of death events by age (in years) for animals that died after 2006. Most deaths correspond to animals that were sacrificed. Colours indicate sex categories (female—light pink, male—light blue, unknown—grey). The x-axis shows the animal age at death, and the y-axis represents the frequency of deaths. Fig. S6: Age-related declines in axolotl mating outcomes vary by sex. **A,** Predicted probabilities (y-axis) of mating outcomes based on female (left) and male (right) age (x-axis) using a multinomial logistic regression model. Mating event is predicted relative to the ‘eggs’ category, chosen as the reference. The model shows how ages affect the likelihood of mating outcome compared to successful event. Hosmer–Lemeshow (HL) test result and McFadden pseudo-R^2^ are indicated. Each point represents the predicted probability for an observation (presence of eggs, no eggs and no sperm, and no eggs). Solid lines represent fitted linear trends for each mating event across age. Colour indicates the mating event. **A′**, Mean predicted probabilities of mating outcomes (y-axis), shown across female and male age groups (x-axis), compared to observed outcomes. **B**, Comparison of male and female age effects on mating outcomes using coefficients from a multinomial logistic regression model. Coefficients represent log-odds of each category relative to the reference ‘eggs’ category. Z-scores (y-axis) were calculated by dividing coefficients by their standard errors. Positive Z-scores reflect an increased likelihood of the category with female or male age (x-axis). Significant results (*p*-value < 0.05) are marked with asterisks. **C**, Chi-squared test for categorical – mating outcome versus age factor – observations in females (left, Χ^2^ = 33.1, *p* = 9.6e-06) and males (right, Χ^2^ = 96, *p* < 2.2e-16), highlighting the significant relationship between mating outcomes and age. Standardised residuals from the Chi-squared test are reported, indicating the strength of deviation from expected mating result for each age group. Positive values (in turquoise) represent higher-than-expected events, negative values (in brown) represent lower-than-expected events. Fig. S7: Regular matings improve larval viability in late-life females. Representative mating history of late life females. The age of female at the time of first and last mating is depicted in the top of the plot. Bars correspond to number of eggs produced and embryos viability, also highlighted with colours. x-axis represents mating number. The average interval between matings in left plot is approx. one month, and at least two months – in the right one. n denotes number of eggs / embryos.

Additional file 3. Table S1. Axolotl colony mating records, including female and male information, date of mating, date of eggs produced, clutch size, and egg quality.

Additional file 4. Table S2. Mating (permanent and not permanent) records of late-life axolotls, including clutch size, fertilised eggs number, embryo number and quality.

## Data Availability

Axolotl mating records are provided in Additional file 3. Mating records of late-life axolotls, including clutch size, embryo number, and quality, are provided in Additional file 4.

## Abbreviations

WT: Wild-type axolotls
DD: White strain of axolotls
HDL: High-density lipoproteins
KO: Knockout
IVF: In vitro fertilisation
PFA: Paraformaldehyde
A-PBS: Amphibian-phosphate-buffered saline
NCAE: Naphthol AS-D chloroacetate esterase
ORO: Oil Red O
KS: Kolmogorov-Smirnov test
HL: Hosmer-Lemeshow test
