# Supplementary figures and images for: Axolotls retain fertility throughout lifespan
Source: BMC Biol. 2026 Feb 14;24:52. doi: 10.1186/s12915-026-02545-3 (PMC12930804; doi:10.1186/s12915-026-02545-3)

**A** Mating statistics, across quarters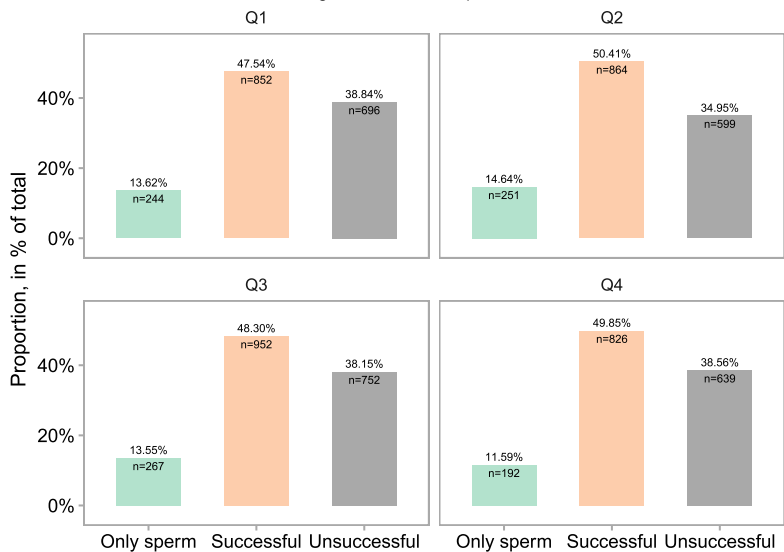**B**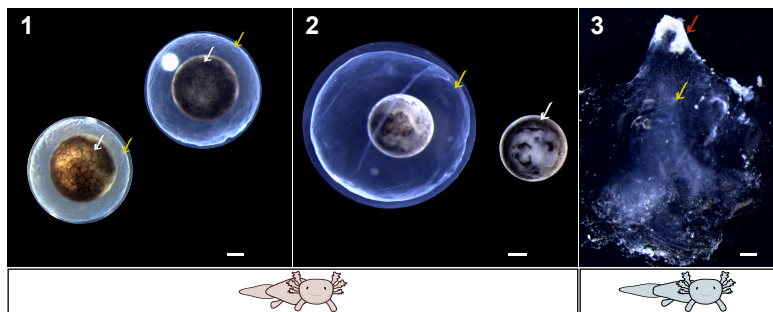**C**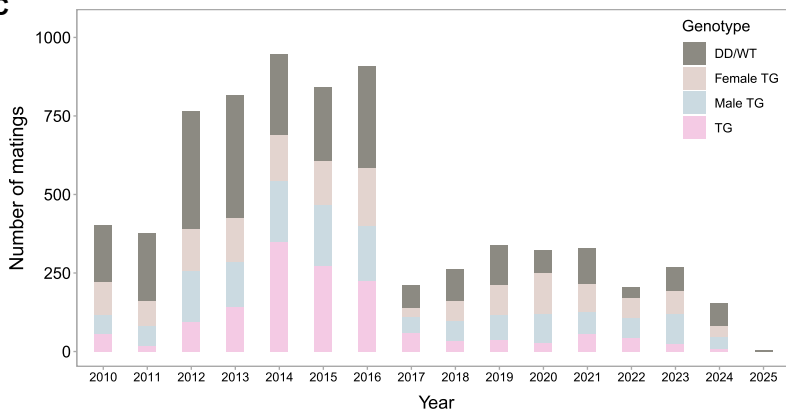

Ovary

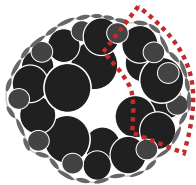

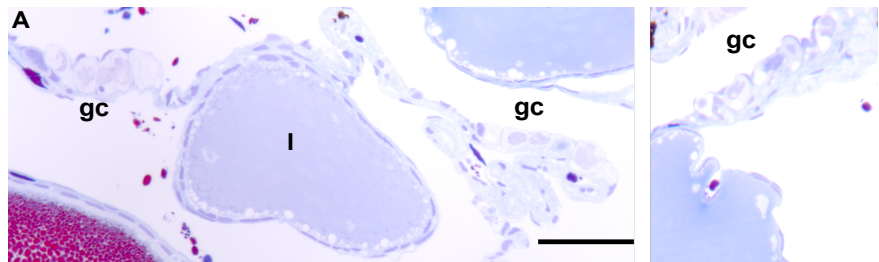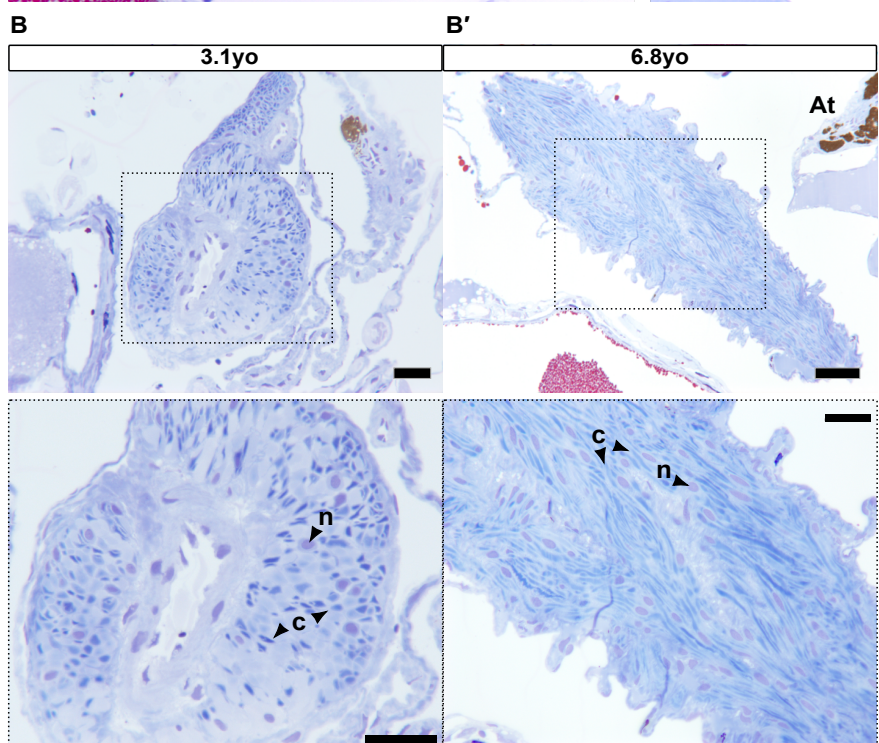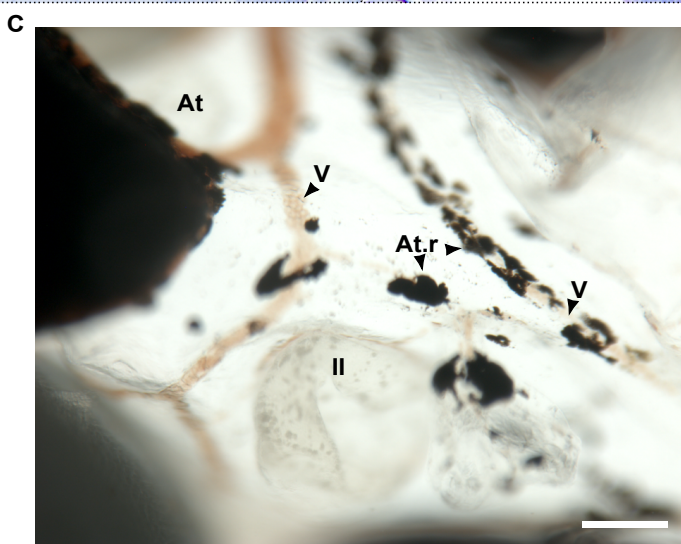

Supplement: Supplementary file 1 — Additional file 1. Figures S1-S3. Fig. S1: Mating success rates remain consistent across different seasons. A, Axolotl colony mating statistics, shown as percentage (y-axis). n denotes the total number of matings. B, Representation of reproductive material: 1, fertilised eggs, 2, unfertilised, necrotic eggs. Arrows: yellow indicates jelly layer, white denotes axolotl embryo; 3, spermatophore. Arrows: red indicates packet of sperm, yellow denotes conical gelatinous base. Scale bar in 500 μm. C, Matings distribution across years between wildtype axolotls and transgenic lines. y-axis denotes the number of matings within respective genotype groups. Fig. S2: Axolotl ovarian tissue collection. Schematic of collection area. Grey dashed lines and red area indicate how samples were collected along the rostro/caudal axis of the ovary. Fig. S3: Histological and whole-mount assessment of axolotl ovarian tissue. A-B′, Mallory trichrome stained sections of axolotl ovary: A and B′ from 6.8-year-old animal, B from 3.1-year-old animal. A, germ cells (gc) based on morphological characterisation, in close proximity to Stage I oocyte. Scale bar 100 μm. B-B′, Visualisation of connective tissue deposition. Collagen (c) fibers (stain blue), nuclei (n). Scale bar in B 50 μm, in B′ upper 100 μm and lower 50 μm. C, Pigmented atretic remnants are located in close proximity to the vasculature. Scale bar 200 μm. Atretic follicle (At), Atretic follicle remnants (At.r), vessels (V), Stage II oocyte (II). [file 12915_2026_2545_MOESM1_ESM.pdf]
