# Supplementary material for: Axolotls retain fertility throughout lifespan
Source: BMC Biol. 2026 Feb 14;24:52. doi: 10.1186/s12915-026-02545-3 (PMC12930804; doi:10.1186/s12915-026-02545-3)
Supplement: Supplementary file 2 — Additional file 2. Figures S4-S7. Fig. S4: Egg size difference with age in axolotls. Axolotl eggs from 1.2-year-old (left) and 3.3-year-old (right) animals. Scale bar 500 μm. Fig. S5: Distribution of axolotl age at death, stratified by sex. The frequency of death events by age (in years) for animals that died after 2006. Most deaths correspond to animals that were sacrificed. Colours indicate sex categories (female—light pink, male—light blue, unknown—grey). The x-axis shows the animal age at death, and the y-axis represents the frequency of deaths. Fig. S6: Age-related declines in axolotl mating outcomes vary by sex. A, Predicted probabilities (y-axis) of mating outcomes based on female (left) and male (right) age (x-axis) using a multinomial logistic regression model. Mating event is predicted relative to the ‘eggs’ category, chosen as the reference. The model shows how ages affect the likelihood of mating outcome compared to successful event. Hosmer–Lemeshow (HL) test result and McFadden pseudo-R2 are indicated. Each point represents the predicted probability for an observation (presence of eggs, no eggs and no sperm, and no eggs). Solid lines represent fitted linear trends for each mating event across age. Colour indicates the mating event. A′, Mean predicted probabilities of mating outcomes (y-axis), shown across female and male age groups (x-axis), compared to observed outcomes. B, Comparison of male and female age effects on mating outcomes using coefficients from a multinomial logistic regression model. Coefficients represent log-odds of each category relative to the reference ‘eggs’ category. Z-scores (y-axis) were calculated by dividing coefficients by their standard errors. Positive Z-scores reflect an increased likelihood of the category with female or male age (x-axis). Significant results (p-value < 0.05) are marked with asterisks. C, Chi-squared test for categorical – mating outcome versus age factor – observations in females (left, Χ2 = 33.1, p = 9.6 [file 12915_2026_2545_MOESM2_ESM.pdf]

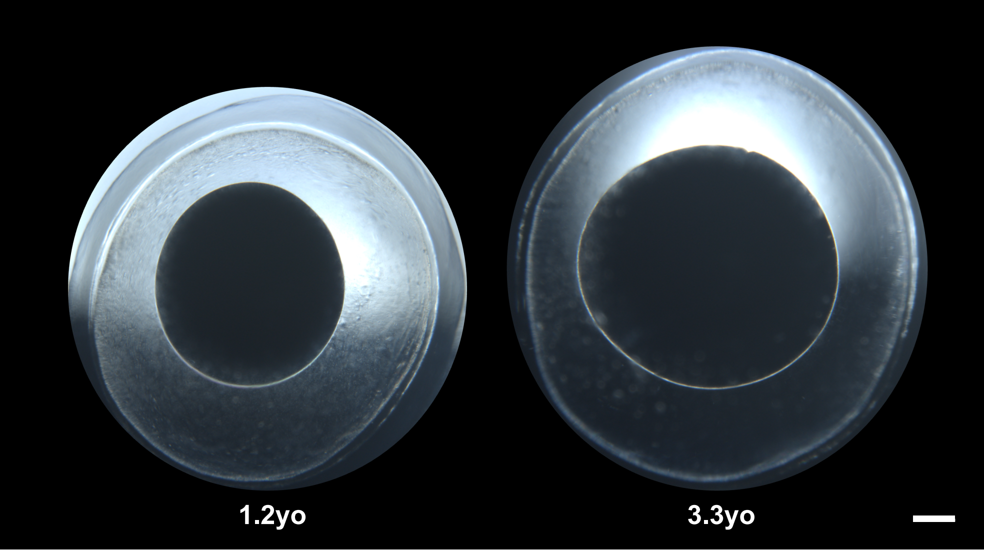

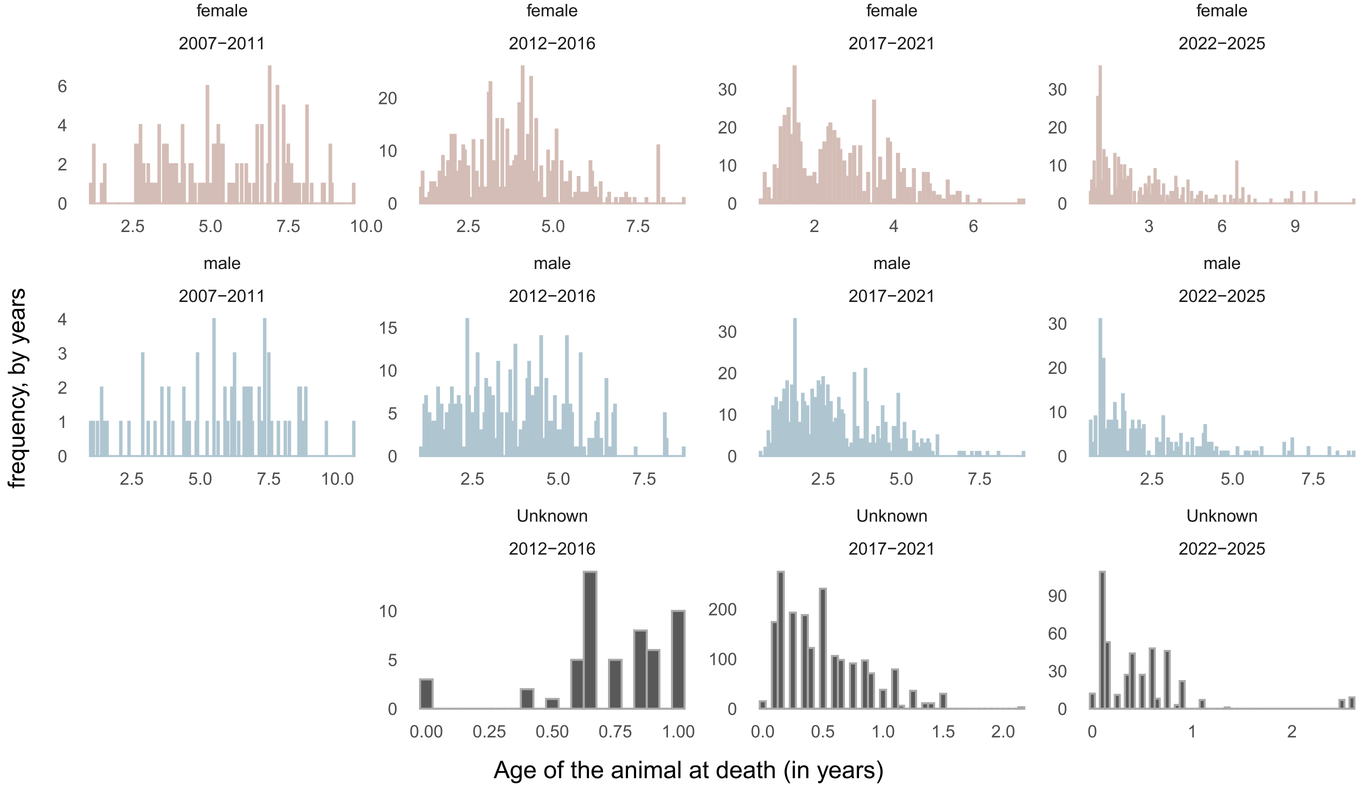
female
2007−2011
female
2012−2016
female
2017−2021
female
2022−2025
male
2007−2011
male
2012−2016
male
2017−2021
male
2022−2025
Unknown
2012−2016
Unknown
2017−2021
Unknown
2022−2025
frequency, by years
Age of the animal at death (in years)

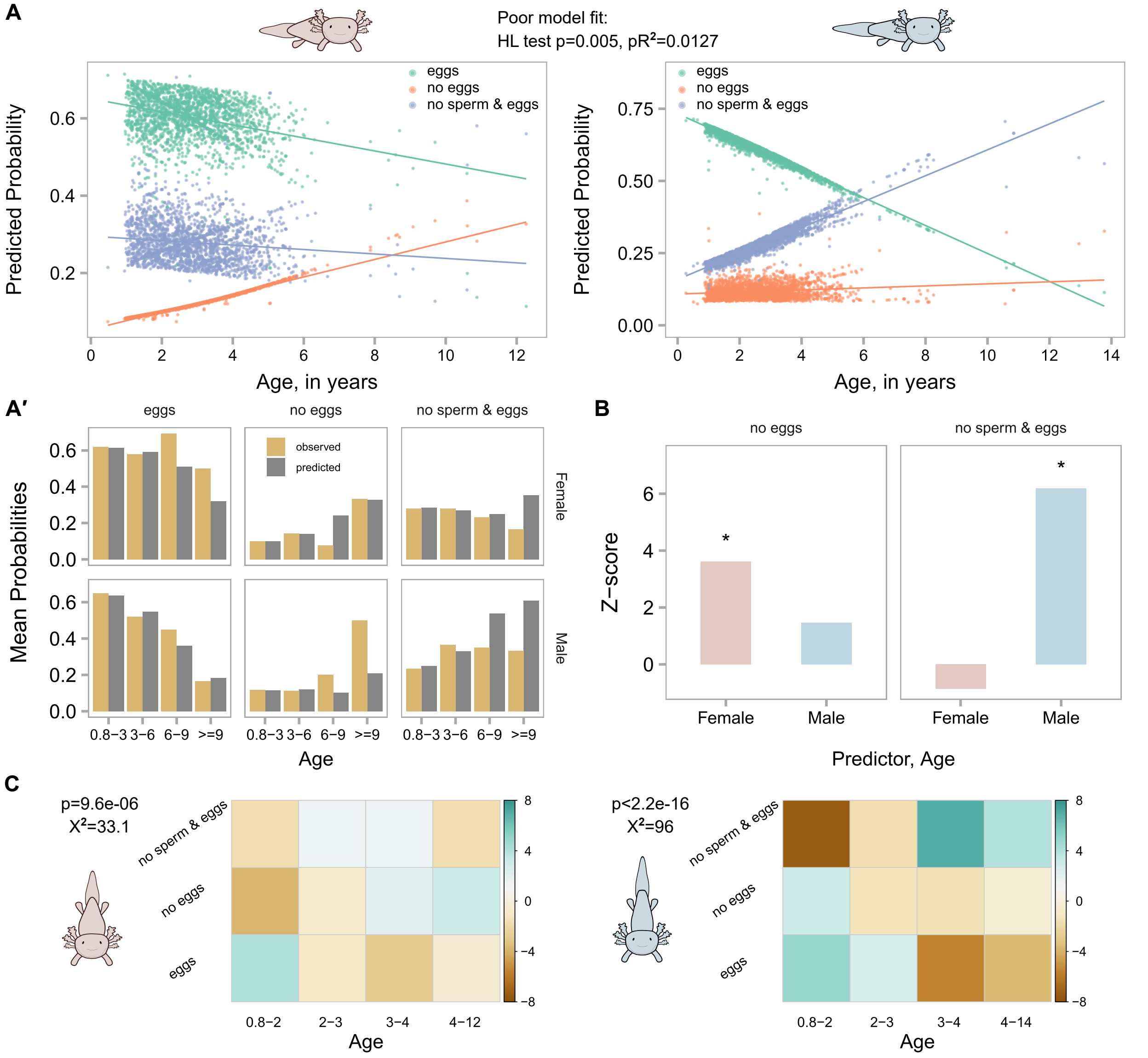
A
Poor model fit:
HL test p=0.005, pR²=0.0127
eggs
no eggs
no sperm & eggs
Predicted Probability
Age, in years
A′
eggs
no eggs
no sperm & eggs
observed
predicted
Female
Male
Mean Probabilities
Age
B
no eggs
no sperm & eggs
Z−score
Female
Male
Predictor, Age
C
p=9.6e-06
X²=33.1
p<2.2e-16
X²=96
no sperm & eggs
no eggs
eggs
0.8−2
2−3
3−4
4−12
4−14
Age

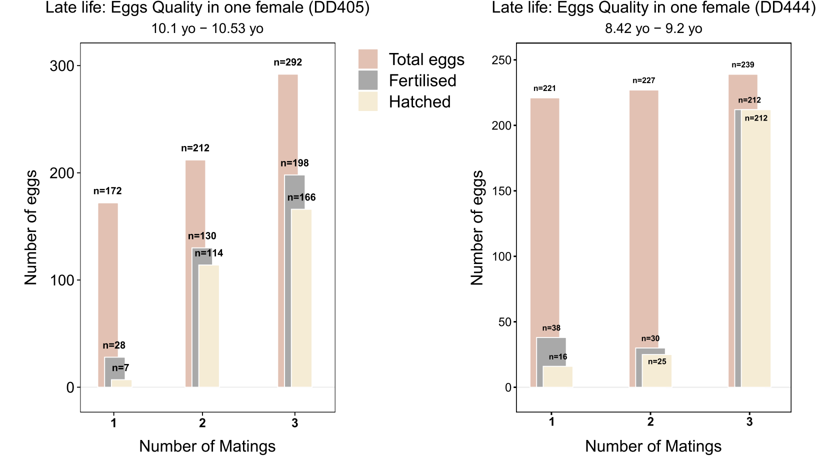

Late life: Eggs Quality in one female (DD405)
10.1 yo − 10.53 yo
Number of eggs
Number of Matings
Total eggs
Fertilised
Hatched
n=172
n=28
n=7
n=212
n=130
n=114
n=292
n=198
n=166
Late life: Eggs Quality in one female (DD444)
8.42 yo − 9.2 yo
Number of eggs
Number of Matings
n=221
n=38
n=16
n=227
n=30
n=25
n=239
n=212
n=212
